# The Influence of Arm Swelling Duration on Shoulder Pathology in Breast Cancer Patients with Lymphedema

**DOI:** 10.1371/journal.pone.0142950

**Published:** 2015-11-16

**Authors:** Dae-Hyun Jang, Min-Wook Kim, Se-Jeong Oh, Jae Min Kim

**Affiliations:** 1 Department of Rehabilitation Medicine, Incheon St. Mary's Hospital, College of Medicine, The Catholic University of Korea, Incheon, Korea; 2 Department of Surgery, Incheon St. Mary's Hospital, College of Medicine, The Catholic University of Korea, Incheon, Korea; Sudbury Regional Hospital, CANADA

## Abstract

**Purpose:**

To evaluate the pathological effect of the duration of arm swelling on the shoulder pathology in patients with breast cancer-related lymphedema.

**Methods:**

Forty seven breast cancer patients with unilateral arm lymphedema were assessed. The duration of the arm swelling and shoulder pain were recorded. Ultrasound examination of the shoulder joint was performed in all patients to detect any lesions.

**Results:**

Abnormalities were detected by ultrasound in 41/47 (87.2%) study participants. Subacromial subdeltoid bursal thickening was found in 26/47 (55.3%) participants, distension of the biceps brachii tendon sheath was found in 14/47 (29.8%) and a supraspinatus tendon tear was found in 13/47 (27.7%). Patients with a supraspinatus tendon tear were found to have a significantly longer duration of lymphedema (1310 days vs. 398 days, *p* = 0.032).

**Conclusions:**

The duration of arm lymphedema has a progressive pathological effect on rotator cuff. Clinicians should adopt an early management approach of shoulder pain in patients with breast cancer-related lymphedema.

## Introduction

Breast cancer-related lymphedema (BCRL) and shoulder pain are common problems and are considered as the quality of life (QOL) predictors in breast cancer patients after surgery [1]. BCRL can cause or aggravate shoulder pain by decreasing the range of motion in the affected limb, increasing the fluid tension in the subcutaneous tissue, increasing the risk of cellulitis and other infections, increasing the risk of lymphangiosarcoma, decreasing the healing capacity in the affected tissue, and causing pathological effects on the rotator cuff tendon [2, 3]. In addition, shoulder pain may potentially cause lymphedema. Breast cancer patients may experience a reduced QOL and a prolonged hospital stay because of these complications [3, 4]. Therefore, an accurate diagnosis and proper management of shoulder pain are necessary for reducing the risk of disability and improving the QOL in patients with BCRL. However, the pathology of the shoulder pain related to BCRL has not yet been fully identified. In addition, BCRL is a chronic condition and the duration of the lymphedema may have an impact on the nature of the shoulder pathology.

A recent report has presented the ultrasound findings for the shoulder pathology in patients with BCRL [5]. However, that study did not stratify patients by duration of BCRL, which prevented any analysis of the cause of shoulder pain as the period after breast cancer treatment. The aim of our present study was to assess the pathological effect of the duration of lymphedema on the shoulder pathology in patients with BCRL by using ultrasound.

## Materials and Methods

### Subjects

This study was conducted with the approval of the Institutional Review Board (IRB No. OC09OISE007) of Incheon St. Mary’s Hospital, the Catholic University of Korea. Written informed consent was obtained from all participants after they had been briefed about the study.

This was a cross-sectional study of 47 women who had been treated for unilateral breast cancer and had subsequently developed BCRL. All participants had access to complex decongestive therapy that comprised manual lymph drainage, compression, exercises, and skin care at our lymphedema clinic. Arm-circumference measurements were taken on both arms at two points- the first point was 10 cm distal to the midpoint of the lateral epicondyle of the humerus and the second point was 10 cm proximal to the midpoint of the lateral epicondyle of the humerus. During the measurements, the participants were prone, with their arms at their sides and elbows straight. The participants who wore compression sleeves removed them 1 hour before the measurements were taken. The mean of two measurements was used as the final measurement [6]. All patients enrolled in this study had an arm circumference difference of > 2 cm above or below the elbow [6, 7], and the diagnosis of BCRL had been previously confirmed by using lymphangioscintigraphy of the upper limbs. All patients were free of cancer at the time of the study. Patients with bilateral lymphedema, lymphangitis, skin disease, inflammatory shoulder arthritis, a previous shoulder joint trauma, and those who had undergone shoulder surgery were excluded from the study.

We recorded the time of onset and the duration of arm swelling (i.e. time from first self-report of symptoms), age, height, and weight of each patient. The intensity of the shoulder pain was measured by using a visual analog scale and each patient completed a disability in the arm, shoulder, and hand (DASH) questionnaire.

### Musculoskeletal Examinations and Ultrasound

Patients underwent a musculoskeletal examination and also an ultrasound of the affected shoulder region. The range of motion of the patients’ shoulder joints was measured to assess the degrees of flexion, abduction, external rotation, and internal rotation, by using a goniometer in the supine position. Ultrasound examination was performed using an HD11 XE Ultrasound system (Philips, Bothell, WA, USA) with a 7–12 MHz line-array transducer. The physiatrist, a Korean board certified physician who was a specialist in musculoskeletal ultrasound medicine conducted the ultrasound examinations, and was blinded to the duration of arm swelling. Each patient was seated on a chair behind the examiner. In accordance with a previously described method [8], five standard ultrasound views (anterior transverse and longitudinal, lateral transverse and longitudinal, and posterior transverse) were selected and a dynamic examination was also included. The subscapularis, supraspinatus, infraspinatus, and biceps tendons were examined in the transverse and longitudinal planes.

The pathologic findings and diagnoses made by ultrasound were based on previously described criteria [9] as follows: a tendon tear was defined as a discontinuity of the tendon fibers that appeared as a hypoechoic or anechoic defect on ultrasound; a partial tear of the tendon fibers involving either the bursal or the articular surface appeared as a focal hypoechoic, or as an anechoic defect on ultrasound. Subacromial subdeltoid (SASD) bursal thickening was defined as a bursal thickness of more than 2 mm transverse with associated hypoechogenicity, with or without bursal fluid. Distention of the biceps brachii tendon sheath was defined as the presence of a hypoechoic or anechoic fluid surrounding the biceps tendon. After a musculoskeletal examination and an ultrasound were performed by the first physiatrist, another physiatrist, a Korean board certified physician, who was a specialist in musculoskeletal ultrasound medicine, confirmed the findings. If there were disagreements about the interpretations about the ultrasound findings between the two physiatrists, then the ultrasound examinations were conducted again.

We also defined adhesive capsulitis as chronic shoulder pain and a loss of 30° or more in the passive range of motion of the glenohumeral joint in external rotation, and at least one of flexion, abduction, or internal rotation.

### Statistical analysis

Statistical analyses were performed with SPSS (version 16.0) software. Data are presented as mean ± standard deviation or the number (percentage) of patients. The primary analysis performed was the comparison of the duration of lymphedema according to the different shoulder pathologies. The Analysis of covariance (ANCOVA) test was used to test for the presence of differences between the groups after adjusting for age. Statistical significance was defined as *p* < 0.05.

## Results

### Clinical characteristics

Characteristics of the patients with BCRL are shown in Table 1. The age range of the patients was 40 to 78 (mean age 52.0 ± 8.5) years. The mean duration of lymphedema was 650.3 ± 1183.9 days. There were 25 patients (53.1%) with ipsilateral pain on the side of the operation. Twenty nine patients (61.7%) had a history of modified radical mastectomy, and 18 patients (38.3%) had a history of partial mastectomy. Forty six patients (97.8%) had a history of axillary lymphadenectomy and 36 patients had undergone radiation therapy. All patients had a history of chemotherapy, 29 patients (61.7%) received chemotherapies including taxanes. Four patients were at stage 3 as per the International society of Lymphology lymphedema staging system, and 43 patients were at stage 2.

**Table 1:** Characteristics of the breast cancer patients with lymphedema analyzed in this study.

Variables	n = 47
Age (years)	52.0 ± 8.9 (40–78)
Body mass index (kg/m^2^)	24.9 ± 3.5
Type of surgery	
Modified radical mastectomy	n = 29 (61.7%)
Breast conserving operation	n = 18 (38.3%)
Axillary lymph node dissection	n = 46 (97.8%)
Radiation Therapy	n = 36 (76.5%)
International society of lymphology stage	
Stage 2	n = 43 (91.5%)
Stage 3	n = 4 (8.5%)
Chemotherapy regimen	
With taxane	n = 29 (61.7%)
Without taxane	n = 18 (38.3%)
Duration of lymphedema (days)	650.3 ± 1183.9
Postoperative days	1191.9 ± 1591.8
DASH	32.3 ± 16.7
Ipsilateral shoulder pain	n = 25 (53.1%)

DASH = disabilities in the arm, shoulder and hand questionnaire.

### Ultrasound findings and clinical features

The abnormal ultrasound findings in the shoulders of the patients are listed in Table 2. Abnormalities were detected by ultrasound in 87.2% (41/47) of all study participants. An SASD bursal thickening was the most common finding in 26/47 (55.3%) patients, distension of the biceps brachii tendon sheath was found in 14/47 (29.8%) patients, and a supraspinatus tendon tear was found in 13/47 (27.7%) patients, respectively. Adhesive capsulitis was found in 11/47 (23.4%) patients. Patients with a supraspinatus tendon tear (n = 13) were found to have a significantly longer duration (1310 days vs. 398 days, *p* = 0.032) of lymphedema. However, the comparisons according to the ipsilateral shoulder pain, SASD bursal thickening, distension of the biceps brachii tendon sheath, and adhesive capsulitis were not statistically significant (Table 3).

**Table 2:** Clinical features of the shoulder disease in the study subjects

Abnormalities of ultrasound findings	41 (87.2%)
Supraspinatus tendon tear	13 (27.7%)
Subacromial subdeltoid bursa thickening	26 (55.3%)
Distension of biceps brachii tendon sheath	14 (29.8%)
Adhesive capsulitis	11 (23.4%)

**Table 3:** The relationship between the duration of lymphedema and shoulder clinical findings.

Clinical shoulder features	Number of patients	Duration of lymphedema (days)	Age adjusted p-value
Ipsilateral shoulder pain			
Yes	25	723.5 ± 1371.8	
No	22	567.2 ± 951.8	0.115
Supraspinatus tendon tear			
Tear	13	1310.3 ± 1713.1	
No tear	34	398.0 ± 805.8	0.032
Subacromial subdeltoid bursa thickening			
Thickening	26	454.8 ± 873.6	
No thickening	21	892.3 ± 1469.2	0.123
Distension of biceps brachii tendon sheath			
Distension	14	909.9 ± 1447.1	
No distension	33	540.2 ± 1059.3	0.168
Adhesive capsulitis			
Yes	11	789.0 ± 1667.1	
No	36	607.9 ± 1019.9	0.183

ANCOVA was used to test for differences between groups after adjusting for age.

## Discussion

Our current study findings demonstrate that the pathology of shoulder pain in patients with BCRL is related to the duration of the lymphedema. We found from our analysis that the duration of lymphedema influences the rotator cuff tendon pathology, but that the symptoms do not correlate with the duration of lymphedema. It is therefore important to determine if there is any correlation between radiological findings and the clinical features of the shoulder disorders among breast cancer patients after therapy.

Several studies have reported various incidences of shoulder pain (from 6% to 68%) in patients surgically treated for breast cancer [10, 11], but few of these reports have assessed patients with BCRL. The incidence of shoulder pain in BCRL cases may be increased because it may cause or aggravate this complication. In our present study, we observed that 53% of our patients with BCRL had ipsilateral shoulder pain, similar to that of a previous study that had reported an incidence of 71% [5]. The incidence of shoulder pain was not related to the duration of lymphedema. We believe that patients with BCRL may have shoulder pain due to different causes, according to the duration of lymphedema. In the early period of lymphedema, patients may be at a high risk for developing shoulder pain because the patient may have undergone adjuvant chemotherapy and/or radiotherapy during this time. Chemotherapy induced fatigue, bone pain, and/or shoulder position for radiotherapy could also cause shoulder pain. Moreover, in the early period of lymphedema, the patients may be more prone to developing shoulder pain due to the inflammatory processes and this could also cause other shoulder pathologies. On the other hand, chronic lymphedema may result in a cumulative load being applied on the rotator cuff muscles and their tendons. Therefore, in the late period of lymphedema, the patients may be at a high risk of developing symptomatic rotator cuff disease [12].

Ultrasound is a useful examination methodology for patients with BCRL. Skin thickness is measured using ultrasound in our clinic, as are the shoulder rotator cuff regions so that appropriate exercise programs can be prescribed. Shoulder exercise programs are one of the components of complex decongestive physiotherapy [13]. Herrera and Stubblefiedl [3] have reported that rotator cuff tendinopathy is a complication of lymphedema caused by an internal disruption to the arrangement of tendon fibers. Mellor et al. [14] have documented that the measurement of skin thickness using ultrasound may be a useful clinical tool for the diagnosis of lymphedema and may also assist with further investigations of therapeutic techniques.

Patients with BCRL commonly present with shoulder discomfort and a previous study [5] has reported that 21.1% of the BCRL cases analyzed had a supraspinatus tendon tear. We found in our present analysis that 27.7% of our patients with BCRL had a supraspinatus tendon tear, which was significantly related to the duration of arm swelling. We believe that lymphedema can affect rotator cuff pathology by causing chronic inflammation and immobilization of the shoulder joint. In addition, longer duration of lymphedema may have a cumulative effect on the rotator cuff pathology through a traction effect, thus providing a possible explanation as to why the supraspinatus tendon tear was related to the duration of lymphedema [12]. In the present study, there was a greater percentage of patients with SASD bursal thickening as compared to a previous study (55.3% vs. 5%) [5]. This disagreement may be caused by the different ultrasound diagnosis criteria used in the two studies. We included the SASD bursal thickening as the pathologic findings of ultrasound, however Jeong et al. [5] used subacromial bursitis as the pathologic findings. It may also explain the discrepancy in the overall rate of abnormal ultrasound findings between the two studies as well.

On the other hand, some of our BCRL patients had complained of contra-lateral shoulder pain also, which may be caused by an overuse of the contra-lateral arm due to a non-use of the BCRL affected arm. As this contra-lateral shoulder could show different pathologic features compared to the BCRL affected arm, further radiologic studies of contra-lateral shoulder pain are required in BCRL patients.

Adhesive capsulitis is also a common finding in BCRL patients. A previous study [5] reported that 21.1% of the BCRL patients analyzed had adhesive capsulitis. Adhesive capsulitis is characterized by pain and stiffness in the shoulder joint, and its prevalence is about 2% in the general population [15]. We found in our current BCRL cohort that 23.4% of the patients had adhesive capsulitis, but that the duration of lymphedema was not significantly significant in these cases.

We also recognize that our present study has certain limitations. First, we assessed a single center cohort with a relatively small number of patients, and did not include a control group. Second, because almost all of the patients were at International Society of Lymphology lymphedema stage II and just two out of the 13 patients with a supraspinatus tendon tear had a full thickness tear, we could not fully investigate the relationship between the severity of the lymphedema and the severity of the shoulder pathology. Further research is recommended in this area. Third, we could not rule out that degenerative rotator cuff pathology could have occurred in some patients regardless of the development of BCRL. Finally, we defined the duration of lymphedema as duration since first self-reports of symptoms. Although early lymphedema can be detected when patients notice changes causing sensations such as swelling and heaviness in the affected limb, it is unclear when lymphedema was diagnosed by objective measures within the study period. And the standardization of measurement and diagnostic criteria of lymphedema were limited. Although the diagnosis of BCRL had been confirmed by using lymphoscintigraphy, we measured both arms circumference only at two points. This method is the most liberal diagnostic criteria for lymphedema at present, and limb volume cannot be calculated from two such points.

In conclusion, our findings suggest that patients who have lymphedema can be affected by a progressive pathological impact on the rotator cuff tendons. We thus recommend that clinicians should adopt an early management approach to shoulder pain in patients with BCRL by making a precise diagnosis of this complication.

## Supporting Information

S1 Table Patient information. (XLSX)

S2 Table Clinical features of the shoulder disease. (XLSX)
